# Pre-Eruptive Intracoronal Resorption: Report of Two Cases

**DOI:** 10.3390/reports9020190

**Published:** 2026-06-17

**Authors:** Līna Petrova, Jūlija Ustiča, Elīna Rasčevska, Shaju Jacob Pulikkotil

**Affiliations:** 1 Faculty of Dentistry, Riga Stradiņš University, LV-1007 Riga, Latvia; 2 Dental Clinic “Zobu Feja”, LV-1011 Riga, Latvia; julijaustica@gmail.com; 3 Shaju Jacob Pulikkotil Oral Health Research Group, Riga Stradiņš University, LV-1007 Riga, Latvia; shajujacob.pulikkotil@rsu.lv; 4 Institute of Stomatology, Riga Stradiņš University, LV-1007 Riga, Latvia; elinarascevska@gmail.com; 5 Department of General Dentistry, Faculty of Dentistry, Riga Stradiņš University, LV-1007 Riga, Latvia

**Keywords:** pre-eruptive intracoronal resorption, permanent dentition, pediatric dentistry, tooth abnormalities

## Abstract

**Background and Clinical Significance:** Pre-eruptive intracoronal resorption is a rare developmental anomaly resembling occlusal caries despite the absence of an external breach. **Case Presentation:** We report of two cases. The first case involves a 9-year-old girl with PEIR of tooth 24 that was not identified on a panoramic radiograph taken one year earlier. The lesion later became clinically evident, presenting with symptoms and discoloration, and progressed to irreversible pulpitis requiring pulpotomy. This case highlights the importance of careful interpretation of paediatric panoramic radiographs and timely intervention to preserve pulp vitality in developing permanent teeth. The second case concerns a 16-year-old girl in whom PEIR was incidentally detected on cone-beam computed tomography (CBCT) in tooth 38. As the tooth has not yet erupted, its future clinical presentation and progression remain uncertain. To the best of the authors’ knowledge, there are no published reports specifically describing PEIR in patients from the Baltic region. **Conclusions:** Early radiographic detection of pre-eruptive intracoronal resorption is essential to prevent pulpal involvement and improve treatment outcomes, particularly when combined with careful interpretation of routine paediatric radiographs to minimize the risk of delayed diagnosis. This case emphasizes the importance of recognizing asymptomatic disease progression and integrating multidisciplinary approach to provide individualized treatment planning.

## 1. Introduction and Clinical Significance

Pre-eruptive intracoronal resorption (PEIR) is an uncommon developmental anomaly characterized by a radiolucent defect within the dentin of an unerupted tooth, typically located just beneath the dentino-enamel junction and resembling occlusal caries despite the absence of an external breach [1,2]. Historically described with terms such as “pre-eruptive caries,” “intrafollicular caries,” and “hidden caries,” current evidence indicates that the lesion represents an externally mediated resorptive process rather than a true carious lesion [3,4]. The term pre-eruptive intracoronal resorption has been used in the literature for many years and remains the most widely recognized terminology for this condition. Although a recently proposed resorption classification would categorize this pathology as pre-eruptive external tooth resorption of the invasive subtype [5], the classification has not yet been formally published. Therefore, the term PEIR was retained in the present report for consistency with the existing literature. The pathogenesis is generally attributed to localized odontoclastic activity originating from the dental follicle or adjacent bone, leading to resorption of coronal dentin before the tooth erupts [1,6].

Epidemiological studies report PEIR to be relatively uncommon but not rare, with subject prevalence ranging from 1% to 4% and tooth prevalence generally below 1% [7]. The condition is most frequently identified in permanent molars and premolars and is usually detected incidentally on panoramic or bitewing radiographs, since the enamel surface typically appears intact before eruption [2,8]. PEIR lesions are frequently overlooked during routine examinations because they are often not visible on standard panoramic radiographs and require careful, attentive radiographic evaluation of unerupted teeth [1,9].

Although some lesions remain stable, others may expand or progress toward the pulp, potentially causing pulpal inflammation or compromising the structural integrity of the crown after eruption [3,6]. The risk of progression is influenced by lesion depth, proximity to the pulp, and stage of root formation [1,6]. There is no standardized treatment protocol, with recommended management varying from periodic monitoring to early surgical exposure, restoration, or vital pulp therapy when pulpal involvement is suspected or confirmed [6,10].

Based on the literature reviewed for this report, we were unable to identify published case reports of PEIR originating from the Baltic region.

Given the diagnostic challenges and the potential consequences of delayed detection, clinical case reports play an important role in illustrating variations in presentation, diagnosis, and management. The present report describes two cases.

## 2. Case Presentation

Written informed consent was obtained from the patient’s parents prior to the preparation of this case report. The study was conducted in accordance with the principles of the Declaration of Helsinki and was approved by the Ethics Committee of Rīga Stradiņš University (Decision No. 2-PEK-4/571/2026, 29 April 2026).

Standardized intraoral photographs and periapical radiographs were obtained (Canon (Tokyo, Japan), Belmont (Sanjō, Japan)) and analysed with dedicated imaging software.

Tooth numbering in this report follows the Fédération Dentaire Internationale (FDI) two-digit notation system.

### 2.1. The First Case

A nine-year-old girl with no relevant medical history presented to the dental clinic in February of 2025 with complaints of tenderness in the maxillary left premolar region during eating. Clinical examination revealed that tooth 24 exhibited a grey discoloration, raising suspicion of internal pathology. The tooth was sensitive to cold with a prolonged response, but it was non-responsive to percussion and palpation (Figure 1).

A review of the patient’s radiographic history revealed that a routine panoramic radiograph taken in January of 2024 had been previously evaluated by the primary dentist and was reported as showing no abnormalities (Figure 2).

However, retrospective evaluation of the panoramic radiograph obtained at the initial visit revealed a previously unrecognized radiolucency within the crown of the unerupted tooth 24. As the tooth had not yet erupted into the oral cavity, the lesion was detectable only radiographically and could not be assessed clinically. The radiolucent defect was located beneath the dentino-enamel junction and extended through most of the dentin thickness toward the pulp chamber. Based on the PEIR classification [2], the lesion was categorized as a Score 3 lesion, as it extended through the full dentin thickness of the crown. The radiographic appearance and its presence before tooth eruption were considered consistent with PEIR. Although changes in the occlusal surface were observed following eruption, which may indicate secondary carious involvement, the presence of the lesion before eruption supports PEIR as the initial pathology.

A subsequent periapical radiograph confirmed the presence of a well-defined intracoronal radiolucency in close proximity to the pulp chamber. No signs of periapical pathology were observed, and root development remained incomplete. Differential diagnoses considered included PEIR with secondary carious involvement, deep dentinal caries, and other developmental intracoronal radiolucencies. Given the radiographic evidence of a lesion present before eruption and its characteristic intracoronal location, PEIR was considered the most likely diagnosis. Due to the extent of the lesion, its close proximity to the pulp, and the symptomatic presentation, the patient was referred to the Riga Stradiņš University Institute of Stomatology for specialized evaluation. During the specialist consultation, the clinical findings were confirmed, including a prolonged response to cold testing, absence of sensitivity to percussion and palpation, and normal periodontal parameters. Based on the patient’s symptoms, the prolonged cold response indicative of persistent pulpal inflammation, and the radiographic finding of a deep intracoronal lesion approximating the pulp chamber, a diagnosis of irreversible pulpitis was established.

In February 2025, a pulpotomy was performed because the tooth exhibited incomplete root development, absence of periapical pathology, and clinical findings suggesting that preservation of radicular pulp vitality was feasible. Following administration of local anaesthesia (1.7 mL, 1:100,000) and rubber dam isolation, peripheral caries was removed to sound dentin margins before pulp exposure. Coronal pulp amputation was completed using a sterile high-speed diamond bur under water cooling. The pulp chamber was irrigated with 2% sodium hypochlorite, and hemostasis was achieved within a few minutes, indicating healthy radicular pulp tissue and confirming the suitability of pulpotomy. The pulp wound was covered with mineral trioxide aggregate (MTA) (MedCem MTA), followed by placement of a glass ionomer cement base. The tooth was restored with a composite restoration involving the mesial, occlusal, distal, buccal, and lingual surfaces.

At the 6-month follow-up visit, the patient reported no pain or other symptoms. Clinical examination revealed no tenderness to percussion or palpation and no signs of swelling or sinus tract formation. Radiographic evaluation demonstrated the absence of periapical pathology and no radiographic signs of treatment failure (Figure 3).

To improve clarity of the longitudinal follow-up, a summary of clinical visits, findings, and interventions is provided in a table format (Table 1).

### 2.2. The Second Case

In February 2026, a 16-year-old girl with no relevant medical history presented for consultation. A cone-beam computed tomography (CBCT) scan was obtained for diagnostic purposes unrelated to the present report. Incidental evaluation of the CBCT revealed a localized radiolucent lesion in tooth 38, consistent with PEIR. At the time of imaging, tooth 38 had not erupted into the oral cavity, and no definite communication between the lesion and the oral environment could be identified on radiographic evaluation (Figure 4).

Given the unerupted status of the tooth, a decision was made to implement close clinical and radiographic follow-up. At present, no specific evidence-based radiographic monitoring interval has been established for unerupted teeth affected by PEIR. Therefore, periodic reassessment is planned, with periapical radiographs taken approximately every 6–12 months for tooth 38, unless earlier imaging is indicated based on clinical or radiographic changes.

Definitive management will be determined following eruption, with the treatment approach—ranging from conservative intervention to extraction—based on lesion extent and potential pulpal involvement. Furthermore, extraction may be indicated due to additional factors, such as orthodontic considerations, and the possibility of eruption failure must also be considered.

## 3. Discussion

The aetiology of PEIR remains unclear and is considered idiopathic. Despite numerous case reports and prevalence studies, the underlying cause of this condition has not been definitively established [1,11].

No significant gender difference has been reported in the prevalence of pre-eruptive intracoronal resorption (*p* = 0.746) [12]; however, in the present report, both patients were female. Molars are most affected, particularly mandibular second molars in paediatric populations and third molars in adults, accounting for up to 59.5% of cases [12,13,14]. In our second case, this pattern is reflected, as the lesion was identified in a mandibular third molar (tooth 38).

PEIR is significantly more prevalent in the mandible than in the maxilla (odds ratio 12.84) [12], while anterior teeth are rarely involved, with central incisors representing only 2.5% of cases [14]. Most lesions are typically located centrally within the crown on the occlusal surface. In contrast to the commonly reported distribution, our first case involved an atypical presentation in a maxillary first premolar (tooth 24), highlighting the variability in lesion location.

PEIR is commonly mistaken for caries, particularly because it resembles dental caries on radiographs and presents as a radiolucent lesion in the coronal dentin [15]. This confusion is so prevalent that PEIR is often referred to as “occult caries,” “hidden caries,” or “pre-eruptive caries” in the literature, reflecting the widespread misdiagnosis.

The radiographic appearance of PEIR closely mimics carious lesions, showing radiolucency in dentin beneath what appears to be an intact occlusal surface [1,15]. The term “occult caries” itself encompasses a range of lesions with prevalence reported from 2.2% to over 50% of permanent molars, but the contribution of PEIR to this overall prevalence remains unclear [1]. When affected teeth are fully erupted, it becomes difficult to determine if pre-eruptive resorption had been present previously, making retrospective diagnosis challenging.

The severity of PEIR lesions can be assessed using several classification systems. One of the most widely used radiographic classifications was proposed by Seow et al. [2], who categorized lesions according to the depth of dentinal involvement: Score 1 lesions extend within the outer one-third of dentin, Score 2 lesions extend up to two-thirds of dentin thickness, and Score 3 lesions involve the full dentin thickness of the crown. In the present report, the lesion in Case 1 was evaluated using periapical radiography and was consistent with a Score 3 lesion, as the radiolucency extended through the entire dentin thickness and approached the pulp chamber. More recently, a new classification was proposed based on a CBCT analysis that allows three-dimensional assessment of lesion extent and location [16]. Because CBCT imaging was available in Case 2, this classification could be applied, and the lesion was categorized as Class 2. The use of these classification systems facilitates standardized description of lesion severity, supports treatment planning, and enables more meaningful comparison of findings across studies.

PEIR continues to present diagnostic and therapeutic challenges due to its subclinical nature before eruption and its tendency to mimic occlusal caries radiographically. Early identification of PEIR is essential because timely intervention significantly improves the prognosis by preventing pulpal involvement and enabling appropriate management of developing permanent teeth [2,3]. In the present case, retrospective examination revealed that the lesion had already been visible on the panoramic radiograph taken a year earlier but was not detected at that time. This underscores the critical importance of careful and systematic interpretation of paediatric panoramic radiographs, as early radiographic diagnosis is the cornerstone for successful management demonstrating the added diagnostic value of three-dimensional imaging, this finding aligns with the broader consensus in maxillofacial radiology that cross-sectional CBCT evaluations consistently reveal anatomical complexities that are often missed on 2D panoramic radiographs. These include bifid and trifid mandibular canals as well as other concealed intraosseous variations in the posterior mandible [17]. The ability to identify such intricate anatomical features further underscores the importance of 3D imaging as the preferred modality for the assessment and follow-up of unerupted teeth in the molar region.

The detection rate of pre-eruptive intracoronal resorption varies markedly depending on the imaging modality used. CBCT has been shown to detect approximately three times more PEIR lesions than panoramic radiography (9.5% vs. 3.1% of patients with unerupted teeth) [14], and when specifically evaluating unerupted teeth, the prevalence identified by CBCT increases to 13.6% [9]. This substantial difference underscores the limitations of conventional two-dimensional imaging and highlights how many lesions may go undetected on panoramic radiographs. Our findings are consistent with the existing literature: in the first case, the PEIR lesion was not recognized on an earlier panoramic radiograph, whereas in the second case, the lesion was identified on CBCT. Although CBCT may provide additional information regarding lesion extent and its relationship to adjacent anatomical structures, its use should not be considered routine for the detection of PEIR. In accordance with current radiological guidelines, CBCT examinations should be performed only when the expected diagnostic benefit outweighs the associated radiation exposure and when the required information cannot be obtained using lower-dose imaging modalities [18,19]. Therefore, careful interpretation of conventional radiographs remains essential for the early detection of PEIR. When a lesion is suspected on two-dimensional imaging and the findings are likely to influence clinical management, CBCT may serve as a valuable adjunct for further characterization and treatment planning [9,14]. In the present report, CBCT was not obtained specifically to investigate PEIR; rather, the lesion in Case 2 was detected incidentally on a scan acquired for an unrelated, clinically justified indication. This approach is consistent with the principles of justification and optimization embodied in the ALARA concept [18,19].

Recent studies have highlighted the value of CBCT in the diagnosis and management of PEIR, providing more accurate assessment of lesion extent, morphology, and pulpal proximity than conventional radiography [9,16]. Lesion progression appears variable, ranging from radiographic stability to progressive enlargement, emphasizing the importance of regular follow-up and individualized risk assessment [20,21].

Current management strategies recommend monitoring small, non-progressive lesions and considering surgical exposure for larger or progressive defects that may compromise pulpal integrity [6,20]. In cases where pulpal exposure occurs in an immature permanent tooth, contemporary evidence supports vital pulp therapy using calcium-silicate biomaterials, such as mineral trioxide aggregate and Biodentine, to preserve pulp vitality and promote continued root development [22,23].

The two cases presented in this report illustrate different clinical scenarios in which PEIR may be encountered. In Case 1, the lesion was visible on a panoramic radiograph obtained before tooth eruption but was not recognized at that time. The delayed diagnosis was followed by clinical symptoms, discoloration, and pulpal involvement, ultimately requiring intervention. In contrast, Case 2 was identified incidentally on CBCT while the tooth remained unerupted and asymptomatic. Together, these cases highlight the variable presentation of PEIR and emphasize the importance of careful radiographic assessment of developing dentition.

Case 1 also demonstrates the value of specialist referral when the diagnosis and treatment plan are uncertain. Following evaluation at the Institute of Stomatology, the clinical and radiographic findings were reviewed and a treatment plan was established based on the developmental stage of the tooth and the extent of the lesion. Although this report does not allow conclusions regarding the superiority of a multidisciplinary approach, it illustrates how additional specialist assessment may assist in the management of complex cases involving immature permanent teeth [6,10].

Because tooth 24 exhibited incomplete root development and no signs of periapical pathology, a conservative treatment approach was selected. Pulpotomy was performed with the aim of preserving radicular pulp vitality and supporting continued root maturation. At the six-month follow-up, the patient remained asymptomatic and no radiographic signs of treatment failure were observed. While the limited follow-up period does not permit conclusions regarding long-term success, the short-term outcome was favourable and is consistent with current recommendations supporting biologically based management of immature permanent teeth whenever feasible [6,10].

The management of Case 2 differed substantially, as the lesion was detected before eruption and no symptoms were present. Consequently, active treatment was deferred in favour of periodic monitoring. This case highlights the clinical uncertainty that may accompany the diagnosis of PEIR in unerupted teeth, as treatment decisions may ultimately depend on eruption status, lesion progression, pulpal involvement, restorability, and other patient-specific factors. Current literature suggests that treatment should be individualized and that extraction should generally be reserved for teeth with poor restorative prognosis or other clear clinical indications [1,7]. The present case supports this contemporary treatment philosophy. Although tooth 24 exhibited grey discoloration and clinical findings consistent with irreversible pulpitis, a conservative approach was favoured because of the biological advantages of preserving pulp vitality and maintaining a developing premolar in the dental arch. Pulpotomy was selected to facilitate continued root maturation and preserve long-term tooth function. At the six-month follow-up, the patient remained asymptomatic, and no radiographic signs of periapical pathology or treatment failure were observed, indicating a favourable short-term outcome. However, given the limited follow-up period, the long-term success of the treatment cannot be confirmed. Nevertheless, this case is consistent with current recommendations advocating biologically based, minimally invasive management of immature permanent teeth whenever clinically feasible.

Although no follow-up data are currently available for Case 2, its inclusion provides an important contrast to Case 1. Whereas the first case illustrates the clinical consequences of delayed recognition of PEIR and subsequent pulpal involvement, the second case demonstrates incidental detection of an asymptomatic lesion in an unerupted tooth using CBCT. This finding highlights the potential value of careful radiographic assessment of developing dentition and underscores the role of CBCT in characterizing lesion extent when imaging is obtained for other clinically justified indications. Furthermore, Case 2 illustrates the clinical uncertainty that may arise when PEIR is identified before eruption, as treatment decisions must balance lesion severity, eruption potential, future orthodontic considerations, and the possibility of spontaneous eruption failure. Therefore, even in the absence of treatment outcomes, this case contributes to the understanding of the diagnostic spectrum and management challenges associated with PEIR.

In summary, early diagnosis, combined with a multidisciplinary approach and the use of modern vital pulp therapy protocols, is essential for achieving optimal outcomes in cases of PEIR. Timely recognition allows clinicians to intervene before extensive pulpal damage occurs, while coordinated specialty care ensures individualized treatment planning that protects pulp vitality and supports normal root development. Advances in materials and treatment guidelines now allow clinicians to preserve teeth that would otherwise have been extracted in the past, strengthening the argument for conservative, evidence-based management of PEIR whenever possible.

## 4. Conclusions

Early radiographic detection of pre-eruptive intracoronal resorption is essential to prevent pulpal involvement and improve treatment outcomes, particularly when combined with careful interpretation of routine paediatric radiographs to minimize the risk of delayed diagnosis. A multidisciplinary approach further enhances accurate assessment and supports individualized treatment planning aimed at preserving the developing tooth. Advances in vital pulp therapy, along with the use of bioactive materials, enable successful maintenance of pulp vitality in immature permanent teeth. Importantly, teeth affected by PEIR generally do not require early extraction unless specific clinical indications arise; instead, a conservative, biologically driven management approach promotes continued root development and supports long-term tooth retention.

## Figures and Tables

**Figure 1:**
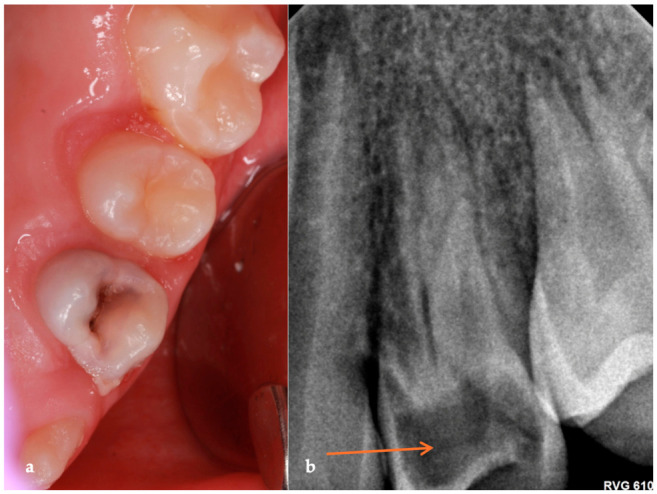
Intraoral photograph and periapical radiograph obtained in February 2025 (patient aged 9 years): (**a**) clinical photograph showing grey discoloration of tooth 24; (**b**) periapical radiograph demonstrating a well-defined intracoronal radiolucency extending through the full dentin thickness of the crown and approaching the pulp chamber. Root development is incomplete, and no signs of periapical pathology are evident. (Please note the area indicated by the orange arrow in the figure.).

**Figure 2:**
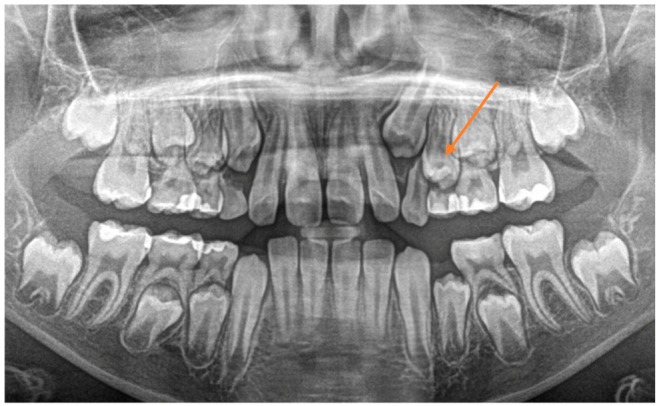
Panoramic radiograph obtained in January 2024 (patient aged 8 years): unerupted tooth 24 demonstrating an intracoronal radiolucency beneath the dentino-enamel junction, extending approximately one-third to two-thirds of the dentin thickness and consistent with pre-eruptive intracoronal resorption. (Please note the area indicated by the orange arrow in the figure.).

**Figure 3:**
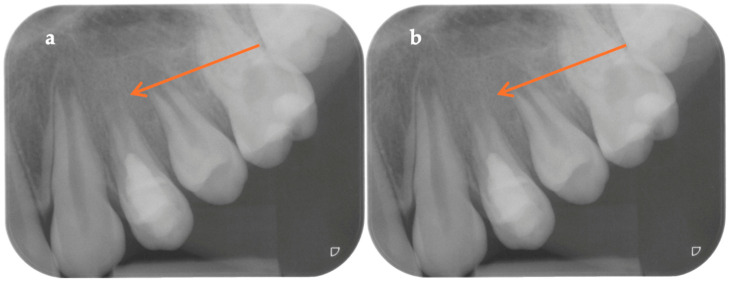
Periapical radiograph (patient aged 9 years): (**a**) periapical radiograph of tooth 24 obtained in February 2025; (**b**) periapical radiograph obtained in August 2025 of tooth 24 demonstrating no signs of periapical pathology (Please note the area indicated by the orange arrow in the figure.).

**Figure 4:**
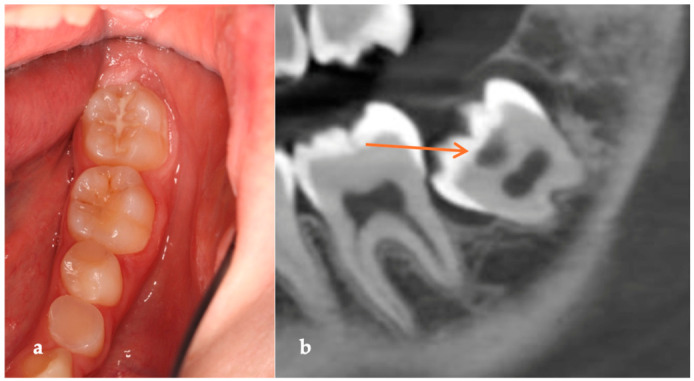
Intraoral photograph and CBCT obtained in February 2026 (patient aged 16 years): (**a**) intraoral photograph of the third quadrant demonstrating that tooth 38 has not erupted into the oral cavity; (**b**) sagittal CBCT image showing a localized intracoronal radiolucency beneath the dentino-enamel junction of tooth 38, extending approximately one-third to two-thirds of the dentin thickness. (Please note the area indicated by the orange arrow in the figure.).

**Table 1:** Summary of clinical visits.

Date	Age	Key Findings	Radiographic Findings	Intervention
January 2024	8 y	No symptoms reported	Panoramic radiograph showed a radiolucent defect within the crown of unerupted tooth 24 (recognized retrospectively)	Lesion not identified at the time of imaging
February 2025	9 y	Tenderness during eating; grey discoloration of tooth 24; prolonged response to cold; negative percussion and palpation tests	Periapical radiograph revealed extensive intracoronal radiolucency and incomplete root development without periapical pathology	Suspected PEIR with irreversible pulpitis; referred for specialist evaluation
February 2025	9 y	Clinical findings confirmed at specialist consultation; periodontal parameters within normal limits	Radiographic findings reviewed and confirmed	Diagnosis of irreversible pulpitis established
February 2025	9 y	-	Roots developed to approximately one-third to two-thirds of their final length	Vital pulp therapy (pulpotomy) performed under local anaesthesia; MTA placement followed by glass ionomer and composite restoration
August 2025	9 y	No pain or other complaints reported	Follow-up periapical radiograph showed absence of periapical pathology and maintenance of treatment outcome	Continued clinical and radiographic monitoring

## Data Availability

The data presented in this study are available on request from the corresponding author. The data are not publicly available due to patient data protection law and confidentiality.

## Abbreviations

The following abbreviations are used in this manuscript:

PEIR	Pre-eruptive intracoronal resorption
CBCT	Cone-beam computed tomography
VPT	Vital pulp therapy
MTA	Mineral trioxide aggregate
ALARA	As Low As Reasonably Achievable
mL	mililiter
